# Microscopic and molecular evaluation of *Strongyloides venezuelensis* in an experimental life cycle using Wistar rats

**DOI:** 10.7705/biomedica.5650

**Published:** 2021-05-31

**Authors:** Jairo Tobar, Daniel Ramos-Sarmiento, Diana Tayupanta, Melina Rodríguez, Fabián Aguilar

**Affiliations:** 1 Área de Parasitología y Bioterios, Gestión de Investigación, Desarrollo e Innovación, Instituto Nacional de Investigación en Salud Pública Leopoldo Izquieta Pérez, Quito, Ecuador Instituto Nacional de Investigación en Salud Pública Leopoldo Izquieta Pérez Quito Ecuador

**Keywords:** Strongyloides, Nematoda, life cycle stages, intestinal diseases, parasitic, Wistar rats, Ecuador, Strongyloides, Nematoda, estadios del ciclo de vida, parasitosis intestinales, ratas Wistar, Ecuador

## Abstract

**Introduction:**

*Strongyloides venezuelensis* is a nematode whose natural host is rats. It is used as a model for the investigation of human strongyloidiasis caused by S. *stercoralis.* The latter is a neglected tropical disease in Ecuador where there are no specific plans to mitigate this parasitic illness.

**Objective:**

To evaluate the stages of *S. venezuelensis* in an experimental life cycle using Wistar rats.

**Materials and methods:**

Male Wistar rats were used to replicate the natural biological cycle of *S. venezuelensis* and describe its morphometric characteristics, as well as its parasitic development. Furthermore, the production of eggs per gram of feces was quantified using two diagnostic techniques and assessment of parasite load: Kato-Katz and qPCR.

**Results:**

Viable larval stages (L_r_ L_2_, L_3_) could be obtained up to 96 hours through fecal culture. Parthenogenetic females were established in the duodenum on the fifth day postinfection. Fertile eggs were observed in the intestinal tissue and fresh feces where the production peak occurred on the 8^th^. day post-infection. Unlike Kato-Katz, qPCR detected parasitic DNA on days not typically reported.

**Conclusions:**

The larval migration of *S. venezuelensis* within the murine host in an experimental environment was equivalent to that described in its natural biological cycle. The Kato-Katz quantitative technique showed to be quick and low-cost, but the qPCR had greater diagnostic precision. This experimental life cycle can be used as a tool for the study of strongyloidiasis or other similar nematodiasis.

## Resumen

**Introducción.:**

*Strongyloides venezuelensis* es un nematodo cuyo huésped natural son las ratas. Se utiliza como modelo para la investigación de la estrongiloidiasis humana producida por *S. stercoralis.* Esta última es una enfermedad tropical desatendida que afecta al Ecuador, donde no existen planes específicos para mitigar esta parasitosis.

**Objetivo.:**

Evaluar experimentalmente los estadios del ciclo de vida de *S. venezuelensis* utilizando ratas Wistar.

**Materiales y métodos.:**

Se emplearon ratas Wistar macho para replicar el ciclo biológico natural de *S. venezuelensis* y describir sus características morfométricas y su desarrollo parasitario. Además, se cuantificó la producción de huevos por gramo de heces mediante dos técnicas de diagnóstico y valoración de carga parasitaria: Kato-Katz y qPCR.

**Resultados.:**

Se obtuvieron estadios larvarios viables (L_1_, L_2_, L_3_) hasta las 96 horas del cultivo fecal. En el duodeno se establecieron hembras partenogenéticas a partir del quinto *día* de la infección. Se observaron huevos fértiles en el tejido intestinal inspeccionado y en las heces frescas, en las que el pico de producción ocurrió al octavo día de la infección. A diferencia del método Kato-Katz, la qPCR detectó ADN parasitario en días que usualmente no se reportan.

**Conclusiones.:**

La migración larvaria de S. *venezuelensis* dentro del ratón en un ambiente experimental fue equivalente al descrito en un ciclo biológico natural. El método cuantitativo de Kato-Katz dio resultados inmediatos a más bajo costo, pero la qPCR tuvo mayor precisión diagnóstica. Este ciclo de vida experimental puede usarse como una herramienta para el estudio de la estrongiloidiasis u otras nematodiasis similares.

*Strongyloides* is a genus of helminths made up of about 50 species of which mainly two, S. *stercoralis* and S. *fulleborni,* are gastrointestinal parasites that may be affecting up to 3,700 million people across the world ^1^^,^^2^. In Latin America, the disease is underestimated due to generally unreliable diagnosis methods. However, there are regions, including Ecuador, that have reported a prevalence between 0,7 % and 4.1 % depending on the ecosystems ^3^^,^^4^. Thus, it is important to acknowledge this parasitic illness as a latent public health risk, especially for inhabitants of underdeveloped areas with precarious health conditions and immunosuppressed patients. For these reasons, strongyloidiasis has been recognized as a tropical neglected disease by the World Health Organization.

Strongyloidiasis is a zoonotic disease produced by *S. stercoralis,* a nematode that lives on the ground as part of its life cycle and can enter the human organism through the skin mainly if the person is barefoot. The parasite has been detected in species such as dogs and primates and it may be accidentally transmitted to humans upon contact with infected faeces ^5^^,^^6^.

The disease may remain asymptomatic for a long time, eventually producing itchiness or hives as the parasite moves under the skin, as well as cough, wheezing, and chronic bronchitis during the infectious stage in lungs or abdominal pain and diarrhea during the intestinal infectious stage. It may also produce autoinfection in the perianal area or in the bowel. The adult larvae penetrate the mucosa, mature, and produce eggs that enter into the lymphatic system and the general circulation and are scattered everywhere in the body where they hatch causing sepsis in various tissues ^7^. The infection may be fatal in cases of immunodeficiency and diagnosis is established through genome amplification in stool samples, serologic tests, and direct microscopy. Ivermectin, thiabendazol, and albendazole are used in the treatment ^8^.

*Strongyloides venezuelensis* and S. *ratti,* which infects rats, have a lifecycle and migration pattern similar to *S. stercoralis,* except they cause no autoinfection and they do not excrete larvae in the faeces. Both parasites exhibit transmammary transmission in different phases. The most notorious difference among both species is that *S. venezuelensis* is less pathogenic than *S. ratti* as a high larval concentration is needed for developing the disease and *S. ratti* larval development is faster, to the point that free living mature females and males may be detected ^9^. Thus, S. *venezuelensis* can be used in inference-based studies to improve the strategies for strongyloidiasis control. A detailed experimental study of the *S. venezuelensis* biological cycle can optimize molecular biology analyses, as well as the knowledge on parasite-host interactions for therapeutic assays to obtain heterologous antigens and develop immunological techniques ^10^^-^^13^.

In this study, we evaluated by microscopic and molecular analyses of *S. venezuelensis* in an experimental life cycle using Wistar rats housed in artificial tropical conditions in an animal facility in Quito, Ecuador. The standardization of this parasite as a model for strongyloidiasis allowed us to describe the morphological and morphometric characteristics of the parasite during its different stages both inside and outside the host. Additionally, we were able to evaluate different diagnostic techniques to detect the parasite in faces and quantify the progress of the infection when evaluating the presence of eggs in stools.

## Materials and methods

### Animals

We used male Wistar rats *(Rattus novergicus)* obtained from Charles River Laboratories, USA, kept in the experimental animal facility at the *Instituto Nacional de Investigación en Salud Pública Dr. Leopoldo Izquieta Pérez* in Quito, Ecuador, at 29°C, 35% humidity, 12/12 light-dark cycle, and 2,850 masl altitude.

The animals selected were 8 weeks old and weighed between 120 and 180 g. Once the experiment ended, the rats were sacrificed using deep intraperitoneal sedation with 10 mg/kg of xylazine (Dormi-Xyl™, 2) and 60 mg/ kg of ketamine (Ket-A-100™) for cervical dislocation.

### Infection of biomodels

Healthy biomodels were inoculated with 3,000 infective L_3_ larvae (iL_3_) in the inner side of the leg subcutaneous tissue. The inoculated larvae's morphology was typically filariform with acceptable motility to light and viability over 95%. The parasites were obtained from the Institute of Biological Sciences at the *Universidade Federal de Minas Gerais,* Brazil.

The rodents were kept in a cage with wood chips, *ad libitum* access to water, and a food ration of 15 g/animal/day. On the 5^th^. day post-infection, the rats were transferred to a metabolic cage. The cage ground had two strips of absorbent paper moistened with distilled water over which a wire net with a 1 cm^2^ aperture was placed to separate the feces from the cage floor and the rats.

### Bronchoalveolar lavage for parasite recovery

For the confirmation of larval migration to the lungs, we used bronchoalveolar lavage in a group of infected animals on the 2^nd^. day postinfection. After euthanasia, dissection was carried out making a 2 cm incision along the middle line in the ventral area of the trachea at one-third cm from the entrance to the thorax. An N° 18 catheter was introduced and fixed with a knot using silk thread.

To develop the bronchoalveolar lavage, 5 ml of phosphate-buffered saline 1X (PBS) with 0.6 mm ethylene-diamine-tetraacetic acid (EDTA) was introduced through the catheter. The fluid obtained was transferred to polypropylene tubes placed on ice. The same procedure was repeated until a total volume of 15 ml was collected, then the tubes were centrifuged at 455g for 15 min at 4°C. The supernatants were discarded and the pellets suspended in 3 ml of RPMI at 4°C to then be transferred to a 24-well culture tissues plate. The cultured plate was observed on the inverted microscope to verify the presence of *S. venezuelensis* larvae.

### Egg counting in feces

From the 5^th^. day post-infection, the feces of the infected rats kept in the cages on dampened paper were collected daily. We took a 5 g previously homogenized sample from the pool of feces to count the number of eggs using the Kato-Katz method with a 41.7 mg template ^14^^,^^15^. The slides were immediately observed with 100X magnification in an optical microscope (Motic, Hong Kong, China) coupled to the Images Plus 2.0™ software (Motic, Hong Kong, China) for the morphometric analyses. The total eggs observed were counted and the final value was multiplied by 24 to calculate de number of eggs per gram of feces.

### Stool culture and larvae maturation

For egg hatching and larval maturing at the first, second, and third stages we used the feces culture obtained from each cage. The feces were mixed with fine-grained vermiculite and sterile water in 28% of the initial stool weight and the culture was then incubated at 28°C.

We collected the larval stages with the modified Baermann technique ^15^^,^^16^. The stool cultures were wrapped in six layers of gauze. Each wrap was suspended over sterile water at 42°C contained in cone-shaped crystal cups and maintained idle for 60 minutes to allow the mobilization of the larvae towards the cup bottom by thermotropism. We eliminated three-fourth parts of the supernatant with a suction pump and one-fourth of the remaining parts was homogenized and transferred to a 10 ml test tube. The tubes were centrifuged for 3 min at 600*g* and the excess liquid was eliminated leaving approximately 2 ml of the supernatant liquid with sediment. The content was homogenized and 2 μl were extended on a slide twice. The slide was visualized at 40X magnification in an optical microscope (Motic, Hong Kong, China) coupled to the Images Plus 2.0™ software (Motic, Hong Kong, China) for the morphometric study.

To study the larval stages, we used the modified Baermann technique 24, 48, 72, and 96 hours after preparing the stool culture. The percentage of larval stages was estimated by visual differentiation and the counting of larvae with motility by calculating the relative frequency percentage for each one of the time periods.

### Analysis of parthenogenetic females

After sacrificing the Wistar rats, we dissected the abdominal cavity, removed the duodenum opening lengthwise, and carefully chopped it. The intestinal tissues were placed over six layers of gauze and the adult larvae were picked up after 3 hours using the modified Baermann method and 0.9% NaCI saline solution as a medium. The resulting fluid was discharged on tissue culture plates to visualize the presence of adult larvae with 40X magnification in an inverted microscope (Motic, Hong Kong, China) coupled to the Images Plus 2.0 software™ (Motic, Hong Kong, China) for their morphological analyses.

### Molecular assays

Feces samples from infected rats were collected in triplicate at the 1^st^., 3^rd^., 4^th^., 5^th^., 7^th^., 8^th^., 11^th^., 15^th^., 21^st^., 28^th^., and 31^st^. days post-infection and conserved in 2.5% potassium dichromate at -80°C until the DNA extraction process. The genomic DNA was extracted using the MagaZorb DNA Mini-Prep Kit™ (Promega, Madison, USA) following the manufacturer's protocols. The DNA was quantified in a NanoDrop2000™ spectrophotometer (Thermo Fisher Scientific, Massachusetts, USA) at 260-280 nm absorbance. As a positive control, we used a sample of S. *venezuelensis* eggs confirmed and quantified by the Kato-Katz method and as a negative control, DNA isolated from the *Ascaris suum, Trichuris trichiura,* and *Taenia saginata* tissues.

We performed qPCR with primers FW5'GAATTCCAAGTAAACGT AAGTCATTAGC-3', RV5TGCCTCTGGATATTGCTCAGTTC-3', and FAMACACACCGGCCGTCGCTGC- BHQ1 to amplify 101 bp of the 18S rRNA region of *S. stercoralis* and *S. venezuelensis*^17^ in a CFX96 Real-Time PCR Detection System Thermal Cycler™ (Bio-Rad, California, USA). Each reaction was prepared in a total volume of 20 μl containing 1 μl of the sample, 10 μl of Master Mix 2X of the GoTaq Probe qPCR Master Mix Kit™ (Promega, Madison, USA), 18 μM of each primer, and 0.8 μM of the FAM fluorophore. The qPCR included an initial denaturation at 95°C for 2 minutes, followed by 40 cycles (95 °C for 3 s, 60°C for 30 s, 72°C for 40 s), and a final elongation step at 72°C for 5 min. The data analysis was carried out using the CFX Manager Software™, version 3.1 (Bio-Rad, California, USA).

The qPCR results were considered negative if the values of the threshold cycle (Ct) were greater than 34 cycles. This value was the detectable limit of the serial dilutions with which the standard qPCR curve was constructed for the molecular quantification of the parasitic load.

### Data analysis

GraphPad Prism software™, version 6.01 (GraphPad Software Inc., California, USA) was used to estimate eggs per gram values on days postinfection and also to calculate the larval stages relative frequency percentage in stool cultures based on incubation hours.

### Ethical considerations

The methodology used was certified and approved by the experimental animal facility of the *Instituto Nacional de Investigación en Salud Pública Dr. Leopoldo Izquieta Pérez.* All the experimental procedures were carried out according to bioethical manuals of experimentation and animal welfare and adjusted to the three R principle, the supervision protocols, the five freedoms principles, and the criteria for humane endpoints, as well as the other recommendations established in the Guide for the Care and Use of Laboratory Animals of the Institute of Laboratory Animal Resources and the National Research Council, USA.

## Results

### Experimental life cycle and detection of the parasite in Wistar rats

**Figure 1:**
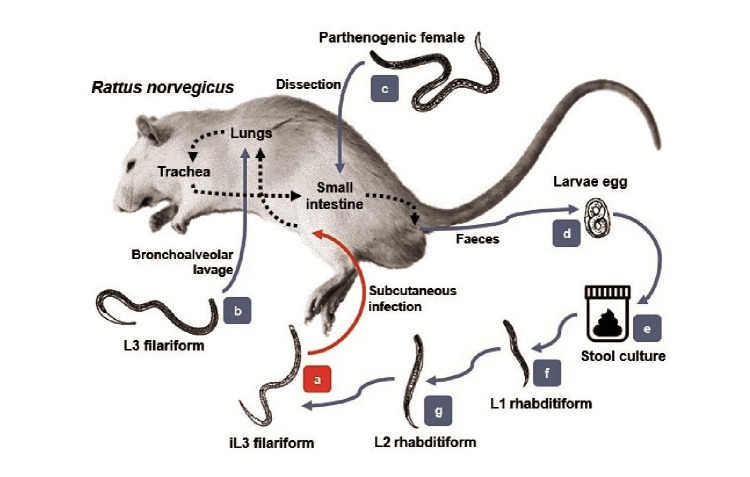
Experimental life cycle of *Strongyloides venezuelensis* in Wistar rats (*Rattis norvegicus)*

After the subcutaneous inoculation to the experimental models, the iL_3_ larvae (figure 1a) migrated through the tissues toward cardiac blood. After the 2^nd^. day post-infection, they were observed in the lungs (figure 1b) with similar morphologic features as those in the infective stage. The larvae then mobilized to the trachea during the next 24 to 48 hours as the animals had been indirectly swallowing them when feeding and hydrating. Afterwards, they migrated to the small intestine on the 5^th^. day post-infection where they matured to the adult form (figure 1c). In this stage, the parasites were hooked on the bowel tissue and the female larvae released many eggs in the stool by parthenogenesis (figure 1d).

**Figure 2:**
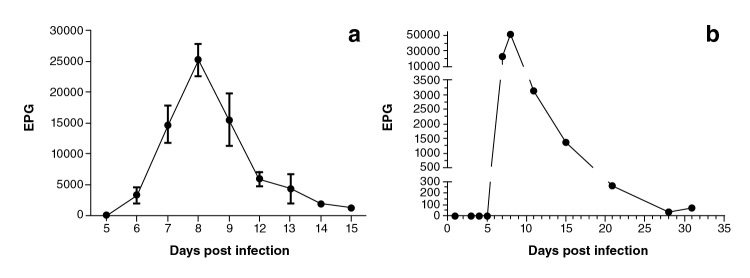
EPG values of *Strongyloides venezuelensis* on the different days post-infection. **a)** Kato-Katz technique analysis (average ± standard error, n=18), **b)** qPCR assay results (EPG: Eggs per gram of feces)

Using the Kato-Katz technique, parasitic eggs were evidenced as of the 6^th^. day post-infection with a peak production on the 8^th^. day post-infection which then descended drastically on the 15^th^. day post-infection (figure 2a) and completely disappeared on the 28^th^. day post-infection. The molecular detection using qPCR allowed to amplify the DNA of the parasite between the 5^th^. and the 31^st^. day post-infection (figure 2b). The egg peak production occurred on the 8^th^. day post-infection according to microscope data. A positive correlation was found (r=0.97) (p-value <0.05) upon correlating the results of calculating the eggs per gram.

**Figure 3:**
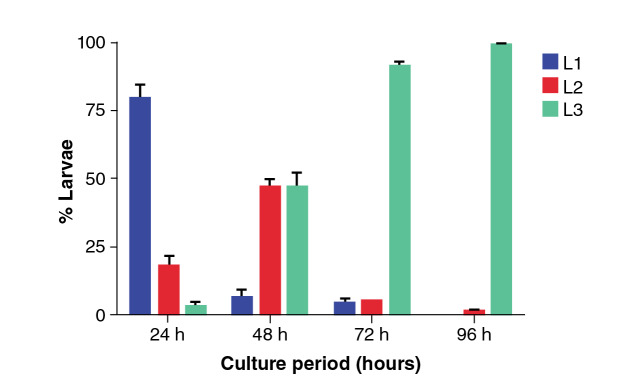
Bar graph of the relative frequency percentage of larval stages of *Strongyloides venezuelensis* in stool culture according to incubation hours

The stool of the infected animals was cultured for egg maturation throughout the different larval stages. As shown in figure 3, within the first 24 hours of incubation it was possible to detect 79% of L_1_ larvae, 18% of L_2_ larvae, and a maximum of 3% of iL_3_ larvae. At 48 hours, 6% of L_1_ larvae, 46% of L_2_, and 48% of iL_3_ were observed. At 72 hours, 4% of L_1_, 5% of L_2_, and 91% of iL_3_ were recovered, and, finally, at 96 hours, 1% of L_2_ and 99% of iL_3_ were found. The best time to recover infective stage larvae was at 96 hours of incubating stool cultures; those larvae were inoculated to preserve the experimental life cycle.

### Eggs morphometric evaluation

**Figure 4:**
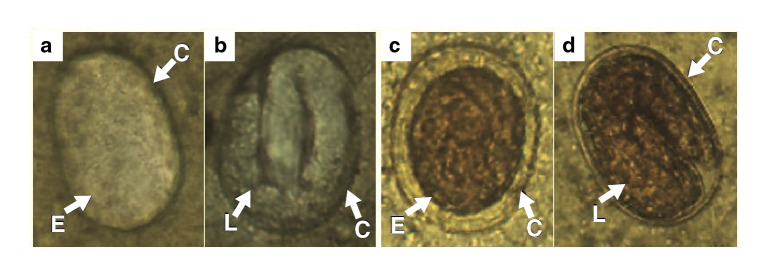
Development stages of *Strongyloides venezuelensis* eggs obtained in feces of infected Wistar rats, **a, c)** Granulated embryo eggs, **b, d)** Larval eggs, **a, b)** Without staining, **c, d)** Lugol staining.

The eggs observed in the feces had different stages of development. In some stool samples, we were able to differentiate granulated embryos (figure 4a, 4c) and larval eggs (figure 4b, 4d) with larva moving inside. Both phases had an oval shape with symmetric polar points and presented a thin chitinous cortex with a smooth surface. Additionally, they (n=92) had an average length of 43.22 urn (standard error=0.23 μm) and a width of 28.8 μm (standard error=0.15 μm).

### Morphometric analysis of larval stages

**Figure 5:**
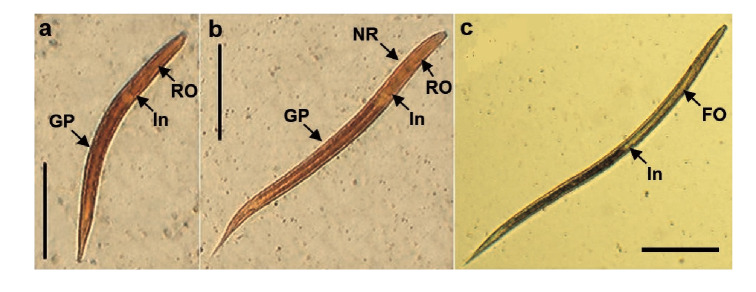
Stages of *Strongyloides venezuelensis* larvae obtained from cultures of infected Wistar rats' stool, **a)** First stage larva *(L*_1_ *).* **b)** Second stage larva (L_2_). **c)** Third stage larva (il_3_) (scale bars: 100 μm).

RO: Rhabdltiform oesophagus; FO: Filariform esophagus; NR: Nervous ring; In: Intestine; GP: Genital prlmordium

The L_1_ larvae (figure 5a) were characterized for having a rounded shape on the front end, a rhabditiform esophagus, and an intestine of approximately half of the total length. Additionally, a genital primordium was present in the central segment and the larvae ended in a pointed tail. They (n=58) were 294.99 μm (standard error=0.23 μm) in length and 17.09 um in width (standard error=0.16 um) in the oesophagus-intestine divide.

The change to L_2_ larvae produced the enlargement of the entire body. They had a rhabditiform esophagus (figure 5b) accounting for approximately 30% of the length of the parasite, which was joined to the intestine, and contained a nervous ring in the central part; a genital primordium was located in the middle ^18^^,^^19^. The L_2_ larvae (n=29) presented a total length of 429.56 μm (standard error=6.85 μm) and a width in the esophagus-intestine division of 17.23 μm (standard error=0.29 μm).

Larvae in the third stage or iL_3_ (figure 5c) presented a rounded front end, a long and filariform esophagus making up for half of the entire parasite dimension. The esophagus was connected to the intestine and ended in a typically sharpened tail. The L_3_ (n=84) had a total length of 547.14 μm (standard error=3.74 μm) and a width in the esophagus-intestine division of 18.54 μm (standard error=0.08 μm).

### Parthenogenetic females' morphometric study

**Figure 6:**
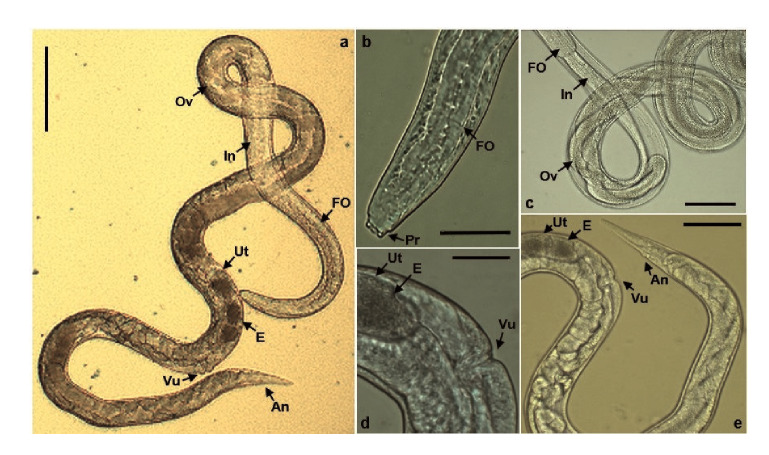
Parthenogenic female of *Strongyloides venezuelensis.* **a)** Entire view (scale bars: 100 μm). **b)** Head portion approach (scale bars: 20 μm). **c)** Middle portion view (scale bars: 50 μm). **d)** Sexual portion approach (scale bars: 20 μm). **e)** Last portion view (scale bars: 50 μm).

FO: Filariform esophagus; In: Intestine; Ov: Ovary; Ut: Uterus; E: Egg; Vu: Vulva; An: Anus; Pr: Projections

Adult parasites were found in the mucus of the small intestine of infected rats. Morphologically, the females (figure 6a) had a rounded front end with chitinous projections like teeth (figure 6b). A cylindrical filariform esophagus was observed, which made up approximately one-third of the body length and was connected to the intestine extending together with the ovary in a spiral shape throughout the parasite (figure 6c). Additionally, the uterus contained granulated embryo eggs along the vulva located on the ventral midline of the parasitic body (figure 6d). The tail was sharp and the anal hole was viewed at one side of its terminal area (Figure 6e). The parthenogenic females (n=49), collected on the 8^th^. day postinfection, had a total length of 2.67 mm (standard error=26.21 μm) and a width at the esophagus-intestine division of 29.81 μm (standard error=0.23 μm).

## Discussion

In this study, we modeled an experimental life cycle of S. *venezuelensis* in Wistar rats maintained on artificial tropical conditions corresponding to the Andean region of Ecuador at 2,850 masl. We were able to observe the biological cycle features, conduct microscopic and molecular diagnoses comparisons, establish morphometric relations, and describe the specific characteristics of the species during the different stages both inside and outside the host.

The methods of parasitic detection we detailed could be used for the diagnosis of homologous parasites such as S. *stercoralis* in humans. From the different qualitative tests, such as direct swabbing and coproparasitic methods with Lugol staining, and the quantitative assays, such as McMaster quantification, serodiagnostic tests (ELISA, IFAT, and immunoblot), or molecular amplification ^10^^,^^11^^,^^20^^-^^22^, we chose the Kato-Katz method as an immediate quantitative measurement technique given that *S. venezuelensis* eggs present an easily degradable thin chitinous membrane common in the Rhabditidae family ^8^^,^^23^. This technique allowed us to observe eggs in the feces as of the 6^th^. day post-infection reaching a maximum peak on the 8^th^. day post-infection with a progressive reduction from the 9^th^. to the 28^th^. day post-infection when none were detected anymore, which agrees with other studies where peaks occurred between the 6^th^. and the 8^th^. day post-infection and their expulsion took less than one month ^10^^,^^11^^,^^22^. The reduction in egg production as of the 9^th^. day post-infection may be attributed to the immune system of the host through the activity of the eosinophils present in the intestine mucosa ^24^, the B lymphocytes ^25^, the mastocytes activated by cytokine stimulus such as IL-3, IL-9, IL-18, and IgE and IgG immunoglobulins ^26^. We did not observe subclinical coinfections with *Syphacia muris,* an oxyurid nematode normally occurring in the gastrointestinal tract of rats, in any of the evaluation days ^22^.

We used qPCR to amplify a specific region of the 18s rRNA gene in the *Strongyloides* genus. Although with the Kato-Katz technique we did not manage to observe eggs on the 5^th^. day post-infection, the results observed by qPCR at this point indicated a small increase (ΔCt=3.68 equal to 1.71 eggs per gram) compared to the negative control. Besides, although the shape of the egg production curve by qPCR was similar to that obtained with the Kato-Katz technique, the concentrations calculated by qPCR were markedly higher, especially on threshold days (7^th^. and 8^th^. day post-infection).

These values could be explained by the different diagnostic potential of microscopical and molecular tests. Moreover, immature parasites could be detected in the small intestine as of 60 hours of infection ^27^^)^ after presumably maturing into adult parasites in a progressive manner, such as that seen for the *in vitro* larvae production. While in this study the 6^th^. day post-infection was not analyzed using qPCR, the microscopic observation of eggs at this point confirmed the presence of the parasite and the establishment of a biological cycle in the infected animals. However, on the last day of the study (31^st^. day post-infection), a small increase was observed in the relative quantity of eggs compared to the previous point (28^th^. day post-infection).

This may have indicated that the adult parasites housed in the intestine slightly increased the oviposition, but not until the necessary limit to be detected by direct microscopy, or that the detected levels could have corresponded to the adult parasites eliminated in the feces, a factor attributed to the immune response that increases the contraction of the intestinal walls favoring their removal ^28^. Given that this study analyzed the dynamic of the biological cycle until the 31^st^. day post-infection, it would be interesting to see the results of the analysis by qPCR after this point to determine the minimum possible amplification levels.

While it is true that the Kato-Katz method was less responsive than the qPCR, its ease of implementation, low cost, and the fact that it does not require sophisticated equipment facilitate its daily use in simple laboratories. Additionally, this technique showed high potential as a screening test for the diagnosis of different nematode infections ^29^^,^^30^^)^ such as schistosomiasis ^15^ and, therefore. it could be used in areas lacking the technological capacity or sufficient resources.

On the other hand, we were able to study the morphologic changes of S. *venezuelensis* stages during the experimental life cycle. *In vivo,* the iL_3_ exhibited the typical migration and penetrated blood vessels after subcutaneous administration ^2^^,^^9^. Then, we found them in lung fluid using bronchoalveolar lavage on the 2^nd^. day post-infection, thus confirming they had reached the alveoli after breaking the capillary membrane and bronchial epithelium, an aggregation that caused small hemorrhages known as pulmonary petechiae ^18^.

Subsequently, they mobilized out of the organism across the trachea to the pharynx where the larvae were swallowed by the animals through food and water. Then, they typically migrated across the digestive tract and matured into adult parasites in the duodenal mucosa and the upper part of the jejunum. The eggs were produced via parthenogenesis by the female larvae and then expelled with the intestinal contents ^2^^,^^9^^,^^31^. Thus, the migration of S. *venezuelensis* was comparable to other parasites of the Secernentea class and similar to other species of the *Strongyloides* genus.

The most important difference between *S. stercoralis* and *S ratti* resided in the capability to develop infective larvae in the large intestine apt to disseminate and create autoinfection and/or hyperinfections in the small intestine or other organs ^7^^,^^9^. *Ex vivo,* the production of larvae *in vitro* was related to the number of eggas per gram in feces and a larger production of larvae on the 8^th^. day post-infection. The larval development was asynchronous before reaching the 96 hours of stool culture incubation due to the progressive maturation of eggs produced by adult females ^20^. At that point, it was possible to find a greater proportion of L_3_ larvae making it an ideal collection point of infective larvae that may be used in different studies.

In conclusion, this study describes the implementation of an experimental model of *S. venezuelensis* in a manageable and reproducible system. The biological cycle we standardized provides a study tool for parasitic biology, toxicology, host-parasite interactions, and the development of new technologies or therapies for strongyloidiasis management or other helminthiasis caused by nematodes of regional importance.
